# Structural characteristics, biomechanics and clinical significance of calcar femorale: A review

**DOI:** 10.1097/MD.0000000000038323

**Published:** 2024-05-24

**Authors:** Xiaoyang Zhou, Junjie Jia, Kai Lian

**Affiliations:** a Department of Orthopedics, Xiangyang No.1 People’s Hospital, Hubei University of Medicine, Xiangyang, China.

**Keywords:** anatomy, biomechanics, calcar femorale, clinical significance

## Abstract

The calcar femorale, first identified by Merkel in 1874, plays a pivotal role in the weight-bearing capacity of the proximal femur, and its structural integrity is crucial for the efficient distribution of mechanical loads. Originating at the vertical ridge where the pubofemoral ligament anchors, this bony prominence extends laterally behind the neutral axis from the medial to lateral aspects. Its presence is not merely an anatomical curiosity but significantly influences the biomechanics of the hip joint by providing additional strength and support against compressive forces encountered during activities such as walking or jumping. Despite its clear description in orthopedic texts, misconceptions persist about its exact function and importance. This article delves into the nuanced anatomy and biomechanical properties of the calcar femorale, offering a detailed literature-based examination that demonstrates its relevance in clinical practice. The review highlights how the robustness of the calcar femorale contributes to the prevention of femoral neck fractures as well as the stabilization of hip prostheses. Furthermore, the indispensable role of the calcar femorale in surgical outcomes is discussed, especially in the context of fracture repair and joint replacement, thus illustrating its enduring significance in contemporary medical applications.

## 1. Introduction

The calcar femorale is a special anatomic location. It starts from the light vertical ridge at the pubofemoral ligament attachment point. It then passes through the rear of the neutral axis from the inside to the outside. The calcar femorale is thickest at the connection of the medial cortical support of the femoral neck and gradually becomes thinner.^[1]^ After total hip arthroplasty, a significant gap exists between the acrylic cement and the cortex of the femoral neck, where the calcar femorale is located.^[2]^ Identifying the calcar femorale is a tortuous process. In 1858, Humphry first described the calcar femorale as “a Cancelli radiating from the back wall of the neck.” In 1874, Merkel used the term femorale calcar for the first time.^[3,4]^ The orthopedic literature has defined the calcar femorale anatomically as “a strong tissue which strengthens the neck of the femur.” However, the terms calcar, calcar femorale, and calcar area are frequently misused. This article describes the anatomy, mechanics, and clinical applications of the calcar femorale based on related literature and discusses the importance of the calcar femorale in clinical treatment.

## 2. Anatomy

**Figure 1.**
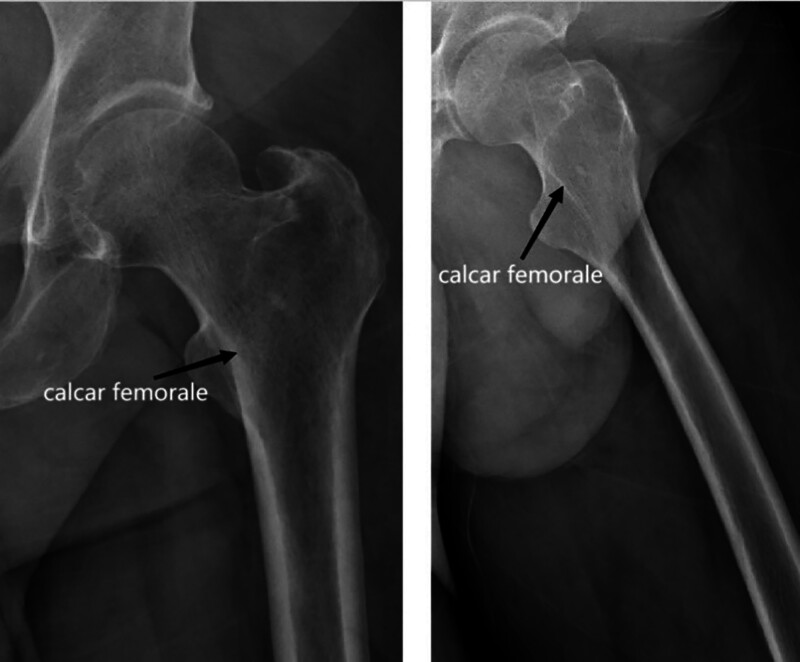
The calcar femorale in X-rays of the proximal femur at different angles.

The calcar femorale is formed by the traction of the iliopsoas muscle. It divides the femoral cortex into 2 layers, the calcar femorale, and the medial femoral cortex. These layers fuse proximally to form the medial neck of the femur (Fig. 1). The calcar femorale is not visible in infant femurs. It develops fully in early adulthood and gradually shrinks after middle age. However, it does not disappear entirely in middle-aged and elderly individuals, as previously believed. Empirical evidence has shown that the lateral cortical thickness and bone mass of the calcar femorale increase with weight and decrease with age in both men and women.^[5]^

Wolff proposed 2 concepts known as the “law of bone remodeling” and “the response of the bone.” These concepts suggest that the structure of a bone is compatible with the biomechanics it is subjected to.^[6,7]^ The calcar femorale, located in the proximal femur, is closely related to its important biomechanical role. On CT cross-sectional images, the calcar femorale can be divided into 3 types based on its size: the ridge, spur, and septum types. The calcar femorale is located deep to and distinct from the lesser trochanter. It is a vertical, quadrilateral plate of dense cancellous bone with an average length of 3 mm, a thickness of 2.71 mm, and a height of 9.94 mm.^[8]^ It starts in the lower part of the vertical trabecular column in the posteromedial section of the femoral shaft and extends to the greater trochanter through the cancellous tissue. The thickest part of the calcar femorale is located at the junction with the medial cortical buttress of the femoral neck.^[9]^ The upper part of the calcar femorale is an extension of the femoral neck that closely connects with the posterior cortex of the femoral neck. It extends far anterior to the trochanter and fuses with the posteromedial side of the diaphysis.^[10]^

The calcar femorale maintains the continuity of the cylindrical femoral shaft, which is disrupted by the formation of the lesser trochanter. It also provides resistance to the compressive stresses in this area.^[11]^ On axial sections, it is transversely oriented in its superior portion. The lower part takes on a more oblique orientation, extending in a posterolateral direction from the medial cortex toward the gluteal tuberosity. As it proceeds inferiorly, its connection with the medial femoral cortex moves anteriorly.

The calcar femorale is not a flat sheet of bone; rather, it has a ribbon-like shape with a slight twist or a spiral portion. The axial direction of the calcar femorale runs parallel to the length of the spur and is perpendicular to the radio direction. Its hardness, density, and mineral content gradually decrease from the outside to the inside.^[12]^

## 3. Biomechanics

The exact function of the calcar femorale in transmitting forces in the proximal part of the femur remains unclear. However, its role is unique and irreplaceable. Farkas et al stated that the calcar femorale is a weight-bearing system at the proximal end of the femur.^[13]^ Li B believed that the calcar femorale provides support for the femoral neck and transmits stress from the trabecular bone of the femoral head and neck to the femoral shaft.^[12]^ Bigelow suggested that the true anterior wall of the upper part of the femur is the calcar femorale.^[10]^ The calcar femorale and the 3 types of bony trabeculae constitute a truss system comprising the compression, oblique, and tension trabeculae. Three types of trabecular bones increase the stiffness of the passage zone by 160% to 400%. The calcar femorale has a higher radiometric density at the proximal femur compared to the predominantly cancellous bone in the rest of the femoral neck. This indicates that it is a significant load-bearing system of the proximal femur, which is consistent with the stress trajectory of the femoral neck. The calcar femorale is capable of withstanding compressive load and redistributing pressure from the femoral head to the proximal femur.^[14,15]^ It forms an angle of 3° to 8° to the perpendicular line and is the only sponge structure in the femoral neck used for weight bearing.^[13]^ Under normal loading conditions, the strain values at the posterior and medial proximal femurs are greater than those at the anterior and lateral femur. The calcar femorale plays a crucial role in redistributing stress by reducing the load in the posterior medial aspects and increasing the load in the anterolateral aspects.^[16]^ If the calcar femorale is damaged, the femoral cortex takes over the role of transferring the load.^[14]^

The stress zone of the proximal femur comprises 4 groups: the primary tensile, secondary tensile, primary compress, and secondary compressive groups. Ward triangle is located in the neutral axis, where there is a balance between the compressive and tensile forces. In the midsagittal image, the compressive trabeculae, visible via 3D CT, are predominantly concentrated on the medial cortex and calcar femorale. In conditions such as coxa vara, coxa valga, or primary bone disease, the calcar femorale bears a larger share of the compressive force. CT reveals increased trabecular bone density in the calcar femorale, underscoring its pivotal role in transmitting proximal femoral force.^[17]^

## 4. Clinical significance of the calcar femorale

### 4.1. The importance of the calcar femorale reconstruction in the internal fixation method

Patients with femoral neck fractures are at high risk of death compared to the general population.^[18]^ The calcar femorale usually reinforces the strength of the femoral neck by resisting torsion. Thus, femoral neck fractures often occur if the calcar femorale is damaged. If a proximal femoral fracture damages the calcar femorale, it is categorized as unstable.

To manage such fractures, achieving anatomic reduction and fixation of the calcar femorale is important. Securing the internal fixation device close to the calcar femorale may strengthen the calcar femorale and preserve the compression force distribution and load transfer at the proximal femur. According to Apel, securing the posterior medial fracture mass, particularly the calcar femorale, is crucial for the mechanical stability of the internal fixators.^[19]^ Surgical placement of fixators aligned with the compression trabeculae and in close proximity to the calcar femorale in treating femoral neck fracture has been associated with favorable outcomes. Levi et al confirmed that the region of highest bone density, as revealed by a CT scan, strongly correlated with the previously reported optimal fixator location.^[20]^ When operating on a femoral fracture with an intact calcar femorale, the internal fixator should be positioned in close proximity to the calcar femorale. This approach maximizes the robust support provided by the calcar femorale while barely disrupting the stress distribution and load conduction in the proximal femur.

**Figure 2.**
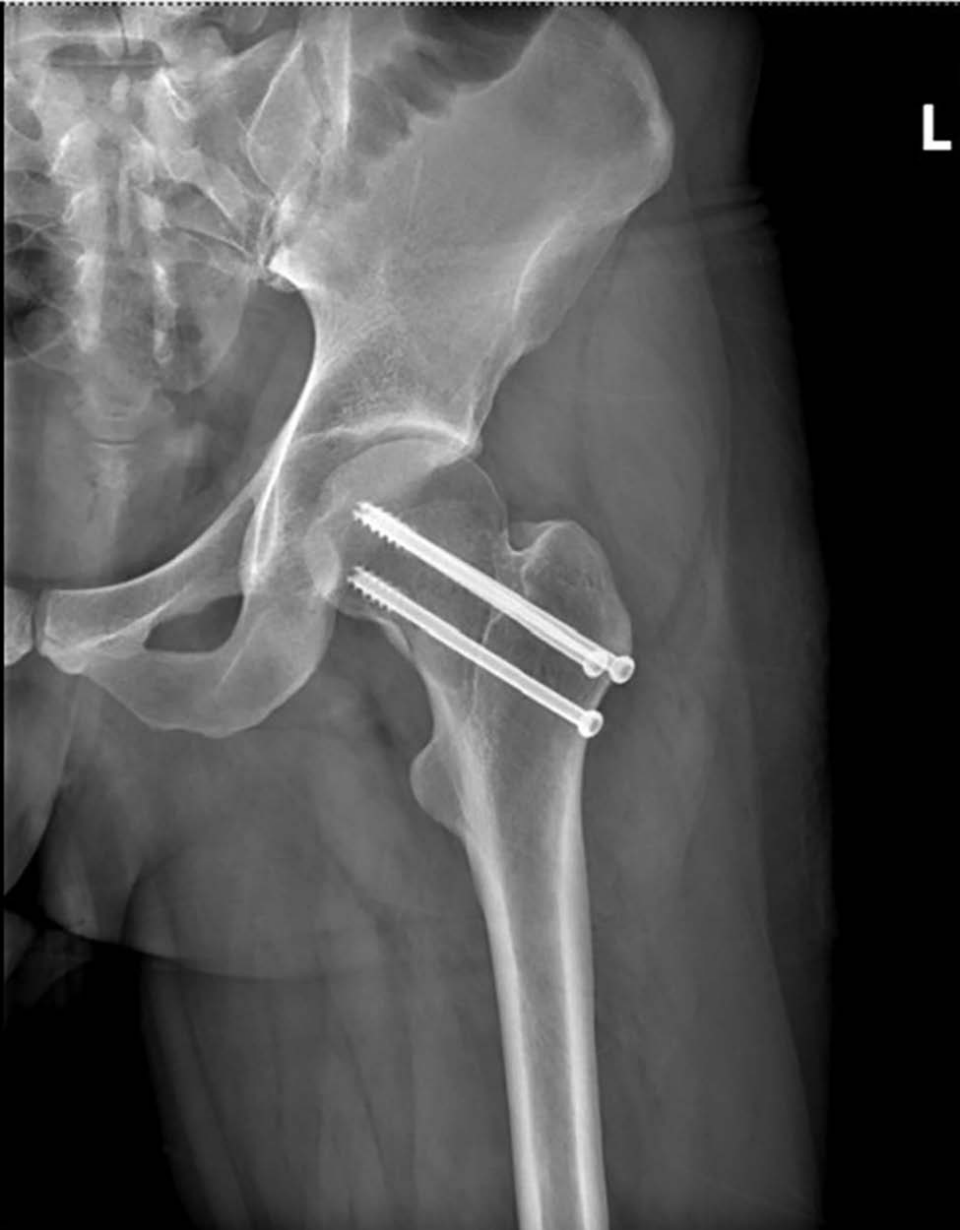
The traditional placement of the cannulated screw in internal fixation of femoral neck fractures.

**Figure 3.**
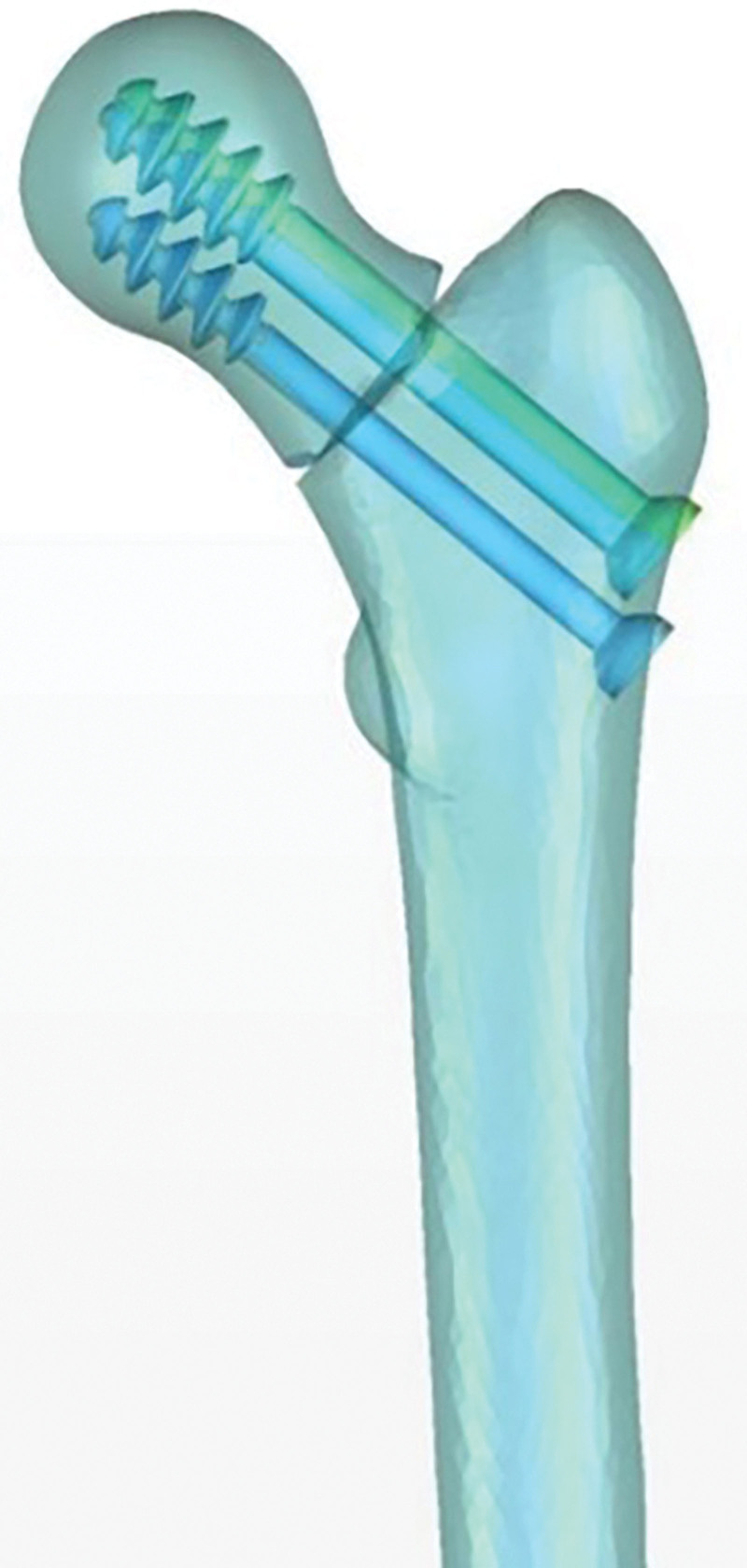
Recommended location for screw placement for femoral neck fracture.

**Figure 4.**
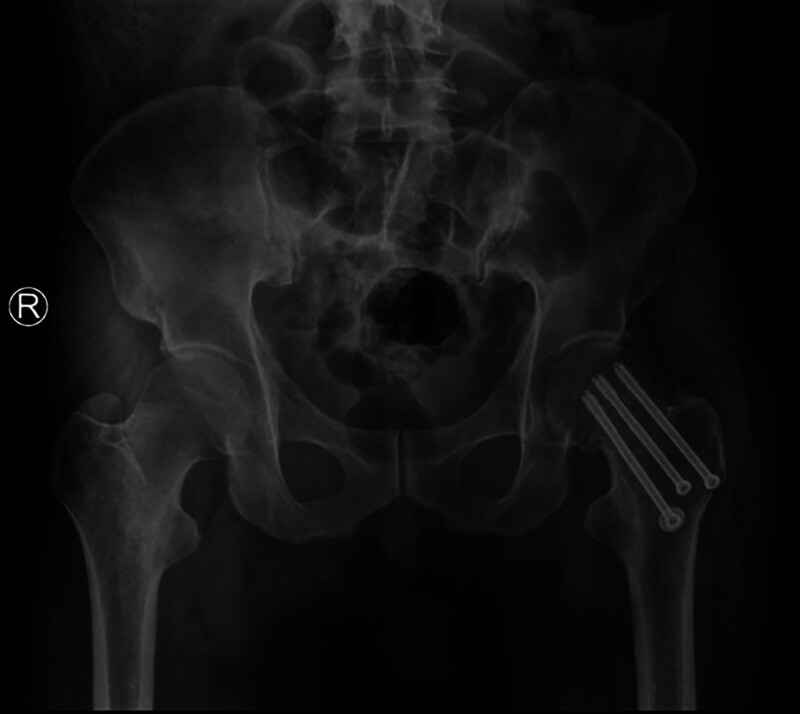
Diagram of divergent screw insertion.

The traditional surgical method for fixing femoral neck fractures involves inserting 3 typical half-threaded screws in an inverted triangle pattern^[21,22]^ (Fig. 2). Yuta Nakanishi indicated that the distal screw should be placed just above the calcar femorale. The anterior screw should be placed 27° anterior to the femoral axis and 14 to 18 mm from the first screw. The posterior screw should be placed slightly posterior to the femoral axis and 10 to 12 mm from the first screw^[23]^ (Fig. 3). Studies have shown that triangular screw fixation in femoral neck fractures provide less displacement, higher peak loading, and more energy absorption before failure.^[24,25]^ Placement of the screws is particularly significant in reducing fractures and preventing bone nonunion.^[26,27]^ However, there is ongoing debate about whether screws should be inserted in parallel. Some studies suggest that parallel insertion screws have a greater risk of causing bone nonunion and osteonecrosis.^[28]^ Ioannis asserted that while parallel screw insertion is not mandatory, it may be more beneficial to place the screw in the biomechanically superior calcar femorale region compared to the traditional inverted triangle implant method. In the traditional method, the first screw is inserted through the middle of the femoral neck, while the second screw is inserted under the first screw at an angle of 20° to 40°. It is important to note that the second screw should be placed on the calcar femorale to provide maximum cortical support. The third screw should be positioned above the first screw and should cross the fracture line (Fig. 4). The final results of the postoperative follow-up indicated that this modified fixation method yielded good clinical outcomes. This is primarily due to the strong support provided by inserting the second screw close to the calcar femorale area.^[1]^ Gao proposed that reconstructing the calcar femorale can effectively reduce complications and improve hip function. This is because the calcar femorale is a crucial supporting structure for the femoral neck. Compared to the traditional inverted triangular nailing approach, a lower cannulated screw placed at an angle of 160° (i.e., close to the angle of the calcar femorale in the inner and distal femoral shaft axis) results in superior peak stress and stress distribution of the fracture ends, femur, and internal fixation. This technique offers better biomechanical performance compared to other approaches.^[29]^

**Figure 5.**
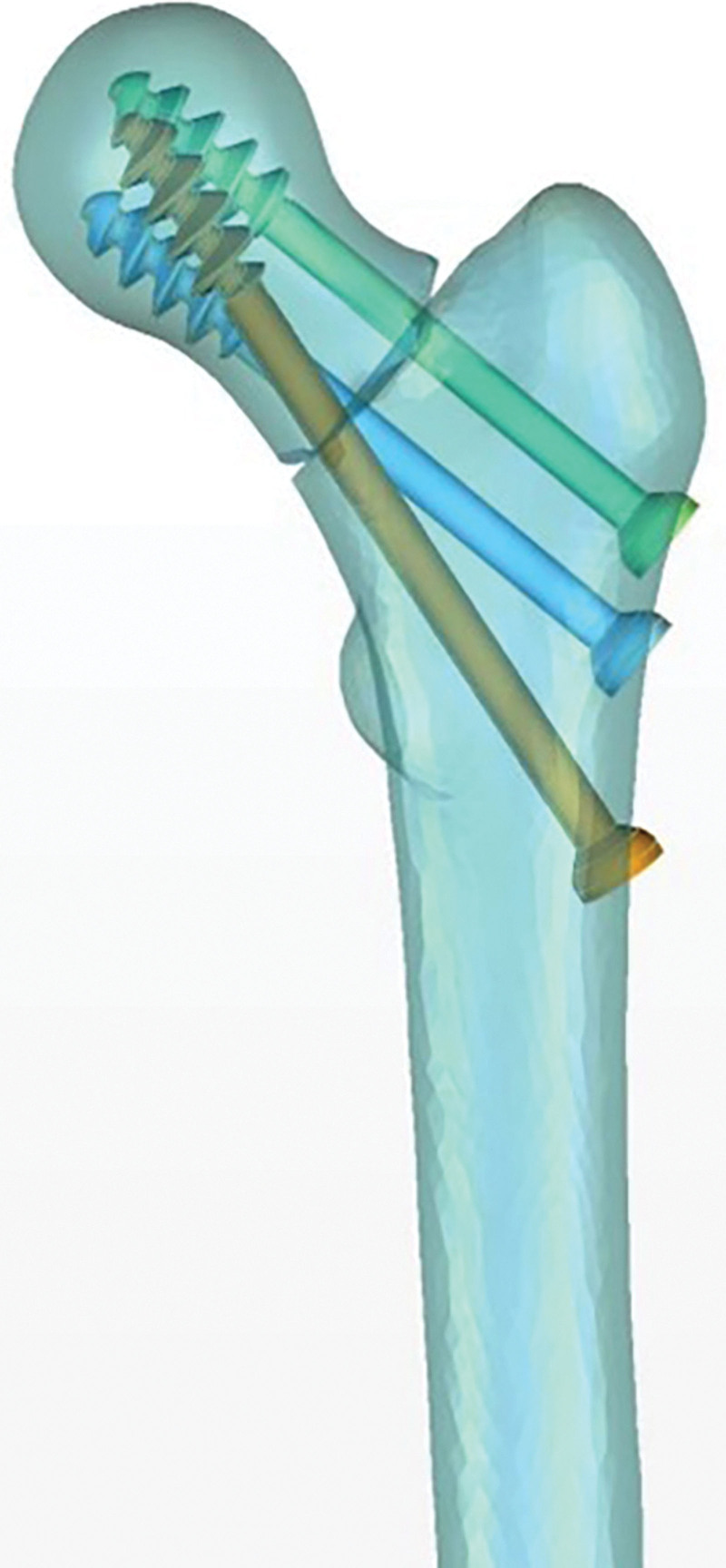
“F” shaped cannulated screw model.

Tianye proposed a new surgical technique for Pauwels type III femoral neck fracture.^[30]^ The procedure involves using 5 to 7 cm of the lower edge of the greater trochanter as the entry point. The first screw is placed at an angle of 150° to 165° to the axis of the backbone, moving from the front to the back. The entry point of the second screw is located 2 to 4 cm from the proximal end of the distal screw, at an angle of 150° to 165° to the backbone axis. The third screw is placed parallel to the middle screw at a distance of 1.5 to 2 cm from its proximal end. After these 3 screws are placed, they form an F-shape (Fig. 5). A steel plate is then placed on the medial side of the femoral neck. Finite element analysis shows that the traditional internal fixation method for femoral neck fractures generates significant stress concentrations in the calcar femorale area. However, the “F” shaped cannulated screw model mainly localizes the stress of the screw at the screw-to-screw joint of the screw head. The stress is then evenly distributed along the screw to the tail of the screw and around it. This covers the discontinuity of the calcar femorale area and transfers the load to the lateral cortical bone, significantly reducing the stress in the calcar femorale area. This helps to enhance the stability of the femoral head under the load and reduces the total displacement. Furthermore, it can maintain the stability of the broken end and create a favorable mechanical environment for fracture healing.

The use of cannulated screws for femoral neck fractures has significantly improved healing rates. However, it has not led to a significant decrease in osteonecrosis of the femoral head (ONFH) and refracture of the femoral neck.^[31]^ ONFH is a devastating disease that can cause collapse of the femoral head and lead to hip arthritis. Necrosis typically affects the weight-bearing area of the femoral head. As the disease progresses, necrotic bones become saponified and mechanically weakened. As a result, stress fractures occur in necrotic bones, which leads to the collapse of the femoral head and subsequent hip arthritis.^[32,33]^ Many surgeons believe that the primary cause of this is biomechanical changes that occur after removal of cannulated screws from the proximal femur.^[34]^ Therefore, the process of implant removal has been receiving increasing attention. Research shows that for the traditional internal fixation method, stress distribution at the proximal femur is similar when only the calcar femorale screw in the lower femur is removed. All other methods lead to stress concentration at the proximal femur, which significantly increases the average stress of the femoral head and femoral neck. When the 2 upper residual screws are extracted at the same time, the average stress of the femoral head and femoral neck is reduced. Conversely, the stress on the screw and the proximal end of the femur is significantly concentrated when the screws are removed separately. Therefore, the best way to remove the internal fixation is to remove the screw closest to the calcar femorale first and then remove the 2 upper residual screws at the same time after the femoral neck has healed. This approach has a minimal effect on the stress concentration at the proximal femur and the average stress on the femoral head.^[35]^

Intertrochanteric fractures (ITFS) are among the most common fractures occurring in older people with osteoporosis.^[36]^ As life expectancy continues to increase, the prevalence of ITFS also increases.^[37]^ About 35% to 40% of these fractures are unstable.^[38,39]^ Hip fractures are associated with higher mortality rates, particularly in older patients. They are typically managed surgically, except in rare cases where the risk of surgical death is considered extremely high.^[40]^ Older patients requiring ITFS revision surgery have a significantly higher mortality and morbidity. Therefore, it is crucial to ensure precise reset, correct fixation, and early mobilization. Intertrochanteric fractures are commonly treated with surgical fixation, as non-surgical treatment can result in prolonged bed rest and immobility. Extramedullary fixation is used for stable fractures, while intramedullary fixation is the preferred treatment for reverse obliquity and unstable fractures.^[41,42]^ Although new technologies and implants are continuously evolving to reduce complications and promote early recovery, failure rates between 0% and 20% remain a concern, particularly for unstable fractures.^[43,44]^ Research shows good outcomes for intramedullary nailing in treating intertrochanteric fractures.^[45]^ Surgical patients may experience complications such as varus collapse, shortening of the femoral neck, and implant failure.^[46]^ Implant design is crucial in reducing complication rates. Studies have demonstrated that implant failure can be influenced by the technical and biomechanical properties of implants.^[47]^ The helical neck blade offers advantages such as anti-varus collapse, stable fixation, and anti-rotation.^[48]^

When using proximal femoral nail anti-rotation (PFNA), it is important to position the blade correctly to avoid major complications such as a perforation of the femoral head. cutout, which is the collapse and varus of the neck-shaft angle, can lead to the extrusion of the lag screw from the femoral head. This is the most destructive complication in the internal fixation of intertrochanteric fractures.^[49]^

This complication is caused by multiple factors, such as bone quality, fracture pattern, implant design, and the position of the lag screw. During the surgical procedure, it is possible to control the position to reduce cutout. Thus, finding the optimal position has been widely discussed in the literature.

**Figure 6.**
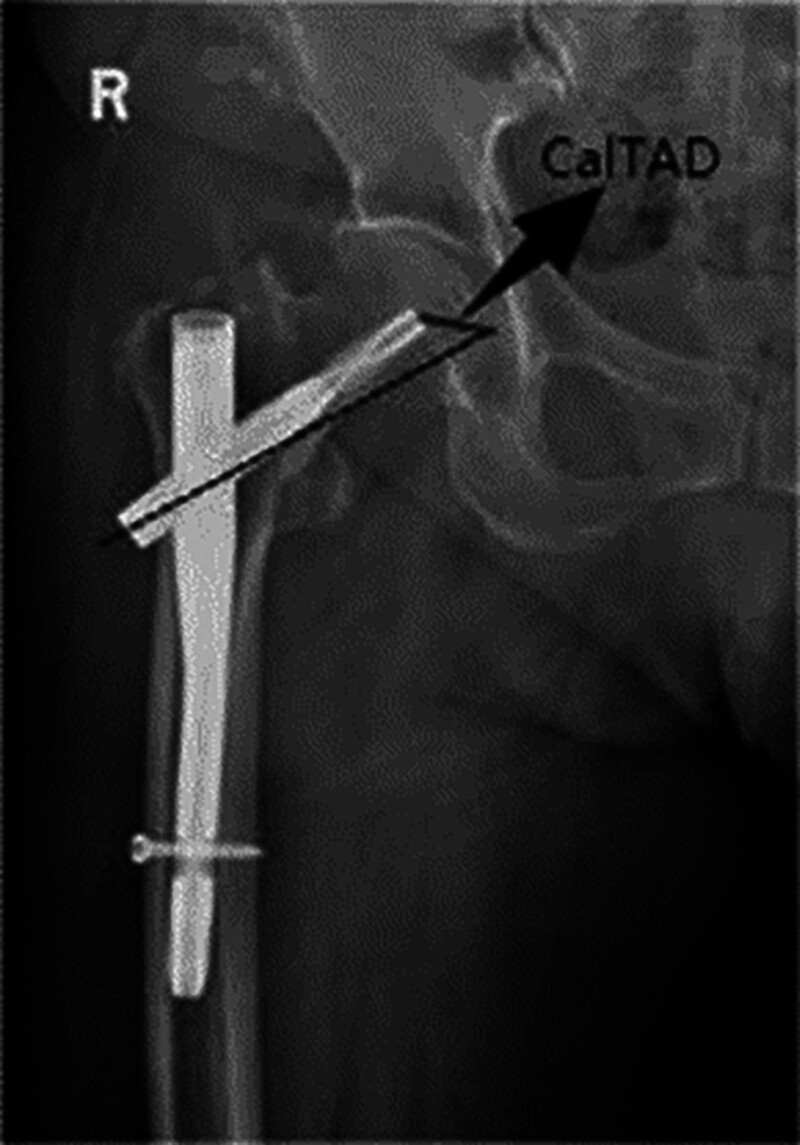
Diagram of calcar-referenced tip-apex distance (CalTAD).

The tip-apex distance (TAD) is the sum of the distances measured on anteroposterior (AP) and lateral radiographs from the tip of the lag screw to the apex of the femoral head.^[50]^ To obtain the AP measurement, the point of intersection between the femoral head and a line drawn just adjacent to the medial cortex of the femoral neck must be determined. This line should be parallel to a guideline drawn through the center of the femoral head and neck. This concept was proposed to link the lag screw position with the risk of cutout and is widely used in clinical practice.^[51,52]^ Kuzyk et al proposed the concept of calcar-referenced tip-apex distance (CalTAD) as a better predictor of cutout risk than TAD. CalTAD is defined as the TAD on the AP radiograph measured with respect to the calcar femorale (Fig. 6).^[53]^

The CalTAD AP is obtained by first identifying the point of intersection between the femoral head and a line drawn just adjacent to the medial cortex of the femoral neck. This line must be parallel to a guideline drawn through the center of the femoral head and neck. The resulting value, measured in millimeters, is then added to the lateral component of the TAD to obtain CalTAD. The AP and lateral positions of the helical blade were measured relative to the femoral head using the AP and lateral Parker ratio index.^[54]^ The values obtained fall within the range of 0 to 100, with lower values indicating a more inferior and posterior position within the femoral head. Stiehl noted that the underside of the femoral neck is a dense trabecular structure that can withstand load stress better than other parts of the femoral neck. During internal fixation, placing the blade on the lower half of the femoral head and the underside of the femoral neck helps achieve stable internal fixation.^[55]^ Jiamton reported a reduced risk of femoral head perforation when the blade tip was located in the inferior half of the femoral head.^[56]^ Kashigar concurred that reducing CalTAD lowers the risk of complications.^[57]^ Compared to the center positioning, placing the lag screw in an inferior position reduces the likelihood of bone volume yielding. Deep placement of the screw tip improves the outcomes of the center and the inferior positions. The best position for the lag screw is placing the screw tip closest to the bone surface in either the center or inferior positions.^[58]^

**Figure 7.**
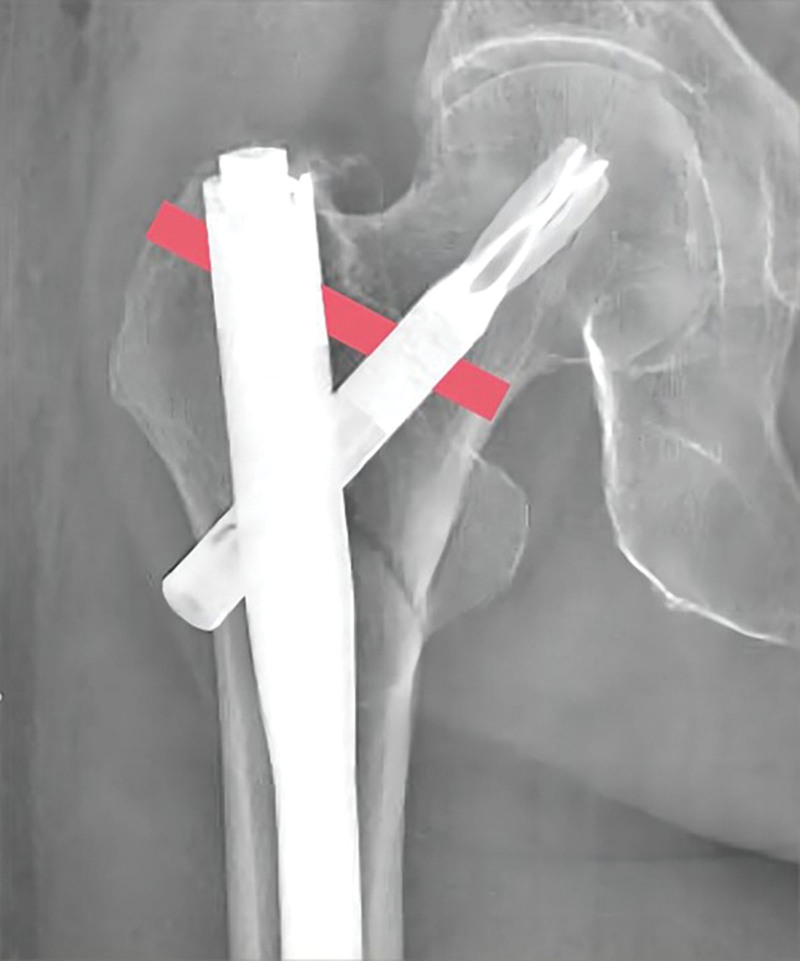
Insertion of additional screw through greater trochanter, nail, and lag screw.

Kerim Öner proposed an improved surgical approach to the existing PFNA system. This was achieved by supplementing the traditional PFNA design with an additional 5 mm diameter screw that passes through the nail, lag screw, and greater trochanter to be positioned on the inner surface of the calcar femorale (Fig. 7). The results of stress analysis revealed that this new procedure reduced the stress level in the calcar femorale location by 30% compared to the standard PFNA procedure.^[59]^

### 4.2. The importance of the calcar femorale in hip replacement

**Figure 8.**
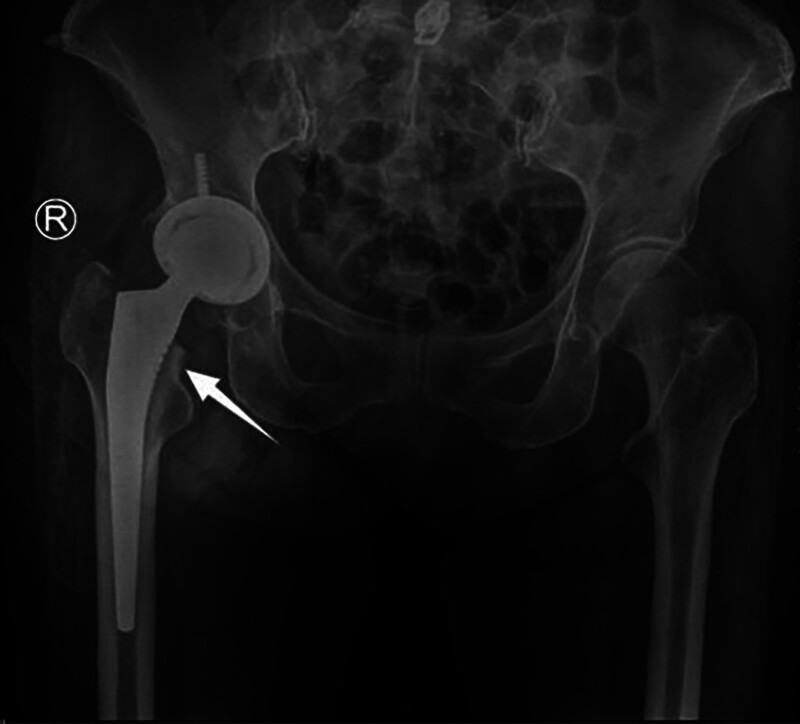
Preserving the calcar femorale during total hip arthroplasty (THA) can achieve a more stable mechanical structure.

**Figure 9.**
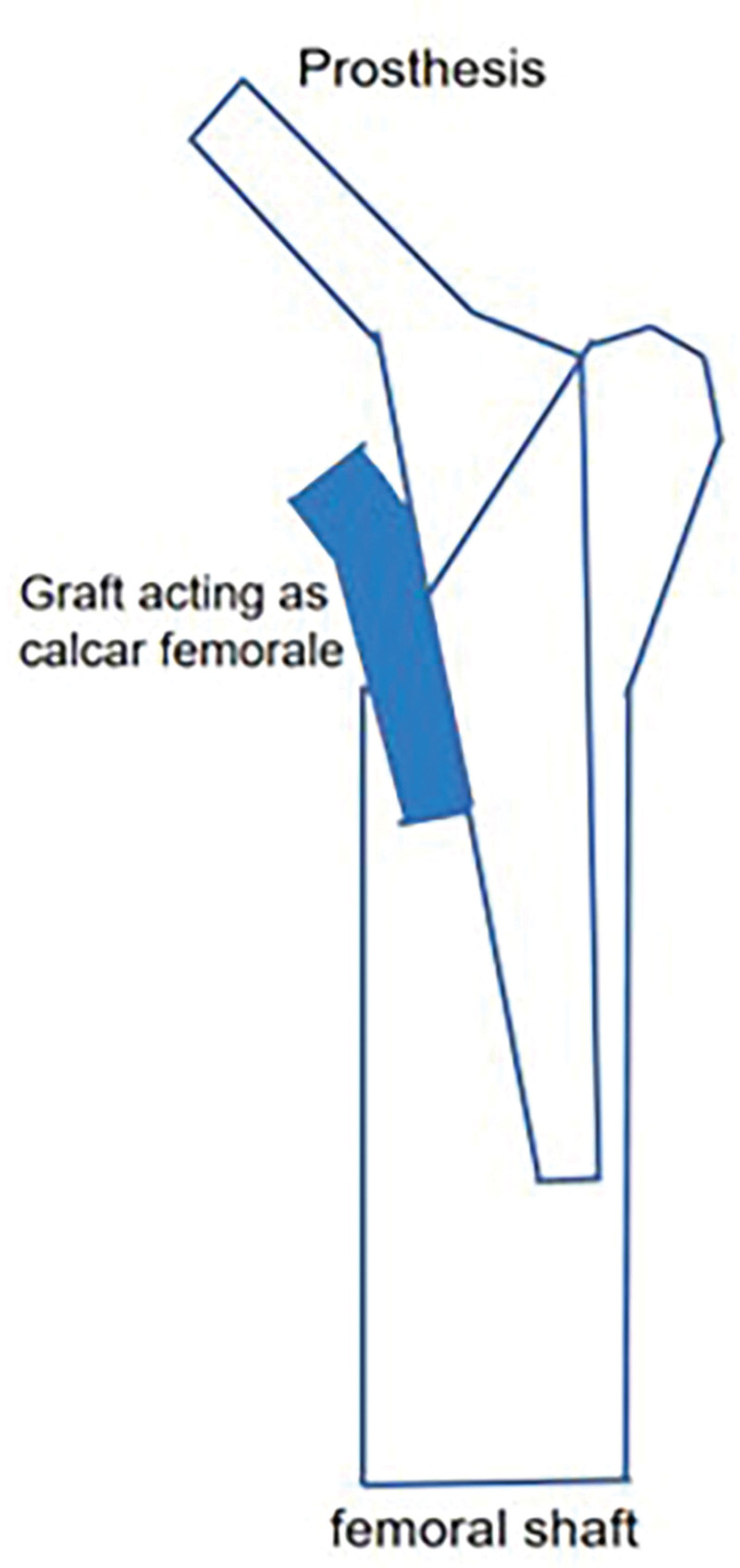
Diagram of the graft wedged into the medial cortex of the proximal femur and the medial edge of the prosthesis.

One of the major postoperative complications of total hip arthroplasty (THA) is aseptic loosening,^[60]^ which occurs due to bone loss and bone resorption around the prosthesis.^[61]^ Preserving or rebuilding the calcar femorale during THA can help establish a more stable mechanical structure (Fig. 8). This has been reported to reduce torsional micromovements between the femoral stems and the host bone after THA. It stabilizes the prosthesis, reduces implant sinking, and maintains postoperative limb length.^[62]^ Thakkar observed that 94% of patients who underwent hemiarthroplasty achieved favorable outcomes after calcar femorale grafting, with minimal complications (Fig. 9).^[63]^ Achieving excellent calcar femorale reduction contributes to satisfactory postoperative results. On the other hand, fractures in the calcar femorale increase the risk of revision after cementless THA.^[64,65]^ Therefore, intraoperative reconstruction of the calcar femorale and prevention of postoperative resorption of the calcar femorale hold significant importance.

Calcar femorale resorption is associated with acrylic cement fixation of the femoral component. Up to 4 mm absorption is considered normal.^[66]^ After femoral prosthesis implantation, the greatest strain occurs near the prosthetic tip rather than at the calcar femorale. When the implant is in place, the pressure in the calcar femorale area diminishes significantly, leading to local disuse atrophy and subsequent resorption after THA. This may increase the risks of bone cement failure, prosthesis loosening, stem failure, and femoral fracture.^[67–71]^

The design optimization of the prosthesis is important to prevent the resorption of the calcar femorale, and this has received widespread attention. The prosthesis design plays a significant role in determining the stress in the calcar femorale area. For prostheses without a medial collar, the stress component within the calcar is usually maximized in the AP direction. The longitudinal compressive stress is less than the tension hoop stress. However, both stress components increase as the length and modulus of elasticity of the stem decrease. The longitudinal stress component of the calcar femorale increases as the cross-sectional size of the stem or the elastic modulus of the cement increases. Adding a stem of the medial collar that is in direct contact with the medial femoral cortex or through the cement layer in the middle greatly increases the longitudinal stress component within the calcar femorale.^[72]^ The load is transferred directly to the calcar femorale via a larger collar in direct contact with the cortical bone. As a result, 30% to 40% of the normal strain of the calcar femorale is restored, reducing the load on the stem and femoral component micromovement. This helps protect the medial cement, medial trabeculae, and stem.^[73]^

Some studies on prosthesis replacement surgery have pointed out that the calcar femorale is as important as the prognosis for choosing surgical methods. Huang noted that the femoral anteversion angle at the center of the lesser trochanter (FA-CLT) level can be defined as the angle created by the line connecting the femoral canal longest transverse diameter and the posterior condylar axis (PCA). Similarly, the calcar femorale angle at the low femoral neck (CF-LFN) level is determined by the angle between the PCA and a line that runs parallel to the calcar femorale. Consequently, the anterior stem anteversion angle of developmental dysplasia of the hip (DDH) patients after prosthesis replacement strongly correlates positively with FA-CLT and CF-LFN. Thus, combining the FA-CLT and CF-LFN could accurately predict postoperative stem anteversion in DDH patients with calcar femorale.^[74–76]^ Accurate prediction of the postoperative stem anteversion allows surgeons to adjust the cup orientation preoperatively and intraoperatively, according to the concept of combined anteversion, enhancing the precision of surgical planning.^[77]^

## 5. Conclusion

The calcar femorale is an important mechanical structure located in the upper femur. It has a unique effect on stress transmission; however, its specific mechanism remains unclear. The calcar femorale redistributes stress; thus, when it is broken, it can lead to increased posteromedial stress. Several studies have found that intraoperative reconstruction of the calcar femorale contributes to improved postoperative efficacy and patient prognosis. Efficient reconstruction of the calcar femorale and minimizing postoperative reabsorption are long-term challenges worth exploring.

## Author contributions

**Conceptualization:** Xiaoyang Zhou, Junjie Jia, Kai Lian.

**Funding acquisition:** Junjie Jia.

**Investigation:** Xiaoyang Zhou.

**Methodology:** Xiaoyang Zhou.

**Project administration:** Xiaoyang Zhou.

**Software:** Xiaoyang Zhou.

**Supervision:** Junjie Jia, Kai Lian.

**Validation:** Kai Lian.

**Visualization:** Xiaoyang Zhou.

**Writing – original draft:** Xiaoyang Zhou.

**Writing – review & editing:** Kai Lian.
